# Safety and Efficacy of VIT against Wasp Venom in Ultra-Rush Protocols in Patients Older Than 60 Years

**DOI:** 10.3390/vaccines12050547

**Published:** 2024-05-16

**Authors:** Andrzej Bożek, Janne Winterstein, Robert Pawłowicz, Ian Poians, Dominika Sadowska, Martyna Miodonska, Marita Nittner-Marszalska

**Affiliations:** 1 Clinical Department of Internal Diseases, Dermatology and Allergology, Medical University of Silesia, 40-055 Katowice, Poland; dominikamangold@gmail.com (D.S.); martynamiodonska298@gmail.com (M.M.); 2 Allergy Outpatient Clinic, Research Department, 80802 Munchen, Germany; jaannewinterstein@gmail.com (J.W.); p.o.j.a.n.ins@wp.pl (I.P.); 3 Department of Internal Medicine, Pneumology and Allergology, Wroclaw Medical University, 50-368 Wroclaw, Poland; robert.pa@wp.pl (R.P.); marmarsz@gmail.com (M.N.-M.)

**Keywords:** venom immunotherapy, insect allergy, immunoaging, basophil activation test

## Abstract

Background: Allergen immunotherapy remains a widely recognized and widely used method for the treatment of selected allergic diseases. Currently, according to the European Academy Of Allergy and Clinical Immunology (EAACI) guidelines, venom immunotherapy (VIT) may be considered for patients over 60. Nevertheless, no separate studies have confirmed the efficacy and safety of this therapy. This study aimed to evaluate the short-term effectiveness of VIT against wasp allergens in an ultra-rush protocol for older patients compared to young patients. Methods: Among the 113 patients included in this study, 51 were older than 60 years (Group A), and 62 formed the control “young group” (age range: 18–35 years). All patients were desensitized to wasp venom using the ultra-rush protocol according to Muller and aqueous solutions of vaccines containing wasp venom. A basophil activation test (Basotest, Orpegen Pharma, Germany) and intracutaneous tests with dilutions of wasp allergen and specific IgE to extract wasp venom were performed at the start and after six months of VIT. The safety of VIT was assessed on the basis of the international Mueller scale. Results: One hundred and eleven patients with confirmed wasp allergies completed six months of VIT: 51 participants over 60 years of age (Group A) and 60 young people (Group B). No systemic adverse reactions were observed during the VIT induction phase. However, large local reactions were noted in 17% of older patients and 20% of young patients at a similar level (*p* > 0.05). During maintenance VIT, two mild grade I systemic reactions were confirmed in young patients. These symptoms resolved spontaneously. There were no such reactions in older patients. The effectiveness of VIT was tested using BAT. There was a statistically significant reduction in CD63 reactivity in 86% of patients in Group A, and a comparable and substantial decrease in 84% of young patients in Group B. According to the BAT test, the mean reductions in the area under the curve (AUC) after six months of VIT were significant (*p* < 0.05) and comparable between Groups A and B: −6.52 vs. 7.21. Conclusions: VIT against wasp venom is safe and effective in short-term observation, and is comparable to that used for young patients.

## 1. Introduction

Allergen immunotherapy (AIT) remains a widely recognized and widely used method for the treatment of selected allergic diseases, such as allergic rhinitis and conjunctivitis; selected forms of controlled or partially controlled allergic asthma; some food allergies (arachidonic nuts); and allergic reactions to Hymenoptera insect venoms [1,2]. Allergen immunotherapy is currently the only causative method for treating allergic diseases and is sometimes a life-saving therapy [1]. Many recommendations, meta-analyses, and recent real-life studies confirm the short- and long-term effectiveness of immunotherapy and its safety, regardless of its administration route [3,4]. For example, immunotherapy for grass allergens in patients with allergic rhinitis and conjunctivitis provides long-term benefits in reducing clinical symptoms and medications during the pollen season and reduces the risk of allergic asthma. AIT has similar advantages for house dust mites and birch allergy. The effectiveness of AIT for other respiratory allergens is less clear, and depends mainly on the individual patient, type of vaccine, and physician experience. It is worth emphasizing that the effectiveness of the described treatment has also been confirmed in patients over 60 years of age and recommended by the EAACI [4]. Regardless, there is a constant need to verify the effectiveness of AIT in specific age groups studied; for example, studies on its effects on children or older people are essential [1,3,5]. Despite the lack of contraindications, not many studies have confirmed the safety and effectiveness of AIT for children and older people [1]. 

VIT is aimed at preventing fatal or life-threatening reactions to stings, while allergen immunotherapy (AIT) aims to reduce or abolish allergy symptoms by inducing tolerance regardless of the recommendation. The effectiveness of such VIT is up to 100% or 95%, depending on whether it is VIT for wasp or bee venom [6]. Most patients achieve this level of protection during the first year of therapy. However, there are no clear data on whether obtaining quick protection using VIT is equally effective in seniors. 

Many recommendations indicate that despite VIT being routinely performed on patients older than 60 years, more data must be collected on the efficacy and safety of VIT while being aware of its necessity as a life-saving therapy [2,6].

The ageing process of the immune system, multiple morbidities including cardiovascular diseases, and the use of multidrug therapies, including beta blockers or converting enzyme inhibitors, may raise understandable concerns about safety and affect patients, especially during intensive immunotherapy protocols such as rush or ultra-rush used for the desensitization to insect venoms. In the available data, older patients have been studied [2,6,7,8,9]. However, these studies on older patients typically focus on those with cardiovascular diseases, and do not form an independent older patient group, and often are not between or above the ages of 60–65 [7,8,9].

Currently, VIT is considered for older people, when they have experienced a severe systemic reaction, provided that they have risk factors such as concomitant vascular diseases, treatment with ACE inhibitors or ß-blockers, severe chronic obstructive pulmonary disease, and reduced quality of life due to previous anaphylactic reactions [2,10].

Pitsios et al. [11] discussed contraindications to VIT; there are no such contraindications to VIT in older patients, and rare severe comorbidities could be a particular limitation. There was no increased risk of adverse effects or increased emergency interventions during VIT in older patients. However, this recommendation was supported only by expert opinions [11]. A short-term evaluation of the effectiveness and safety of VIT in seniors is very important because it will increase the qualifications of these patients for treatment. Due to the lack of such evidence, some patients over 60 are still disqualified from VIT for fear of side effects, especially in rapid protocols [11].

The aim of this study was to evaluate the effectiveness of VIT against wasp allergens in an ultra-rush protocol for older patients compared to young patients. At the same time, it was an attempt to assess the rapid effect of VIT and whether the aging process of the immune system affects such effectiveness. For this purpose, the BAT test was used as the only recommended tool for such an assessment. This study also analyzed whether the commonly recommended ultra-rush VIT protocol is as safe for older patients as it is for younger age groups. Two different wasp sting vaccines were used to obtain more reliable results.

## 2. Materials and Methods

### 2.1. Study Design

This multicenter, prospective, interventional, open study compared the study group (A) to the control group (B) according to age and compared between subgroups (A1, A2, B1, and B2) according to age and type of intervention. This study was registered in ClinicalTrial.Gov (NCT03157505) [12]

### 2.2. Patients

**Figure 1:**
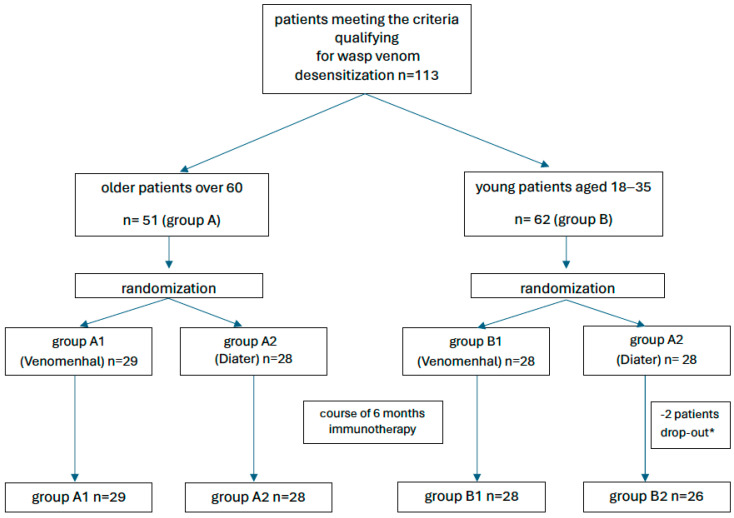
Diagram flow. Legend: * 2 patients drop out due to new contraindications to continue VIT.

In order to reliably assess the safety and efficacy of VIT in elderly people, the study design assumed a comparison of a group of seniors with a group of younger patients but with the same characteristics of a wasp sting allergy. Among the 113 patients included in this study, 51 were older than 60 years (Group A), and 62 formed the control “young group” (age range: 18–35). In every group, patients were randomized to receive the Venomenhal or Diater vaccination at a ratio of 1:1. The randomization procedure used computer-generated numbers (Microsoft, Redmond, WA, USA). This resulted in the formation of four subgroups: A1, elderly individuals receiving Venomenhal; and A2, elderly individuals receiving Diater; B1, young individuals receiving Venomenhal; and B2, young individuals receiving Diater (see Figure 1). A total of 111 patients completed the first 6 months of immunotherapy and were included in the final analysis. The characteristics of the patients are presented in Table 1.

The inclusion criteria were as follows: aged between 18 and 35 years or older than 60 years; confirmed allergy to wasp venom (IDT, sIgE); and indications for VIT according to the EAACI criteria (previous systemic grade III or IV reactions according to the Mueller scale or grade II reactions at the express request of the patient). The exclusion criteria were lack of informed consent, acute infections, unstable chronic disease that temporarily excluded the patient from VIT during recruitment to this study, pregnancy at the time of qualification for VIT, and active cancer where the benefits of VIT were questionable.

This protocol was approved by the ethics committee of the Medical University of Silesia and German Medical Chamber Munich, and all patients provided informed consent.

### 2.3. Diagnostic Procedures

#### 2.3.1. IDT

Intracutaneous tests were performed using the following concentrations of the prepared venom extracts: 0.01 µg/mL, 0.1 µg/mL, 1.0 µg/mL, 10.0 µg/mL, and 100 µg/mL. Extracts of wasp venom obtained from the Venomenhal or Diater vaccines and solutions were obtained based on the manufacturer’s instructions. The results are expressed according to the scale proposed by Saint-Laudy et al. [13] The results were as follows: 0 = negative at 100 µg/mL, 1 = positive at 100 µg/mL, 2 = positive at 10 µg/mL, 3 = positive at 1 µg/mL, 4 = positive at 0.1 µg/mL, and 5 = positive at 0.01 µg/mL.

#### 2.3.2. Specific IgE (sIgE)

The sIgE to wasp venom extract values were determined by UniCap (Thermo Fisher Scientific, Waltham, MA, USA), and the results were expressed according to the manufacturer’s recommendations. Additionally, the levels of IgE components in rVes v1 and rVes v5 were determined using the same method. The cut-off point for a positive result is >0.35 kU/L. All IgE tests were performed only during VIT qualification.

#### 2.3.3. Basophil Activation Test (BAT)

BAT was performed according to the manufacturer’s protocol (Basotest, Orpegen Pharma, Germany) to assess the effectiveness of VIT after 6 months of therapy. Briefly, 100 μL of heparinized blood sample was incubated with 20 μL of stimulation buffer for 20 min at 37.5 °C, and then, standard allergen wasp venom extract was added at concentrations of 0.001, 0.01, 0.1, and 1 μg/mL, and a positive or negative control was added. Basophil degranulation was finished by chilling on ice for 5 min. Then, the cells were stained with anti-IgE/phycoerythrin as well as anti-CD63/fluorescein isothiocyanate monoclonal antibodies. Finally, basophil activation was analyzed using a FACSCanto II system (BD Biosciences, Erembodegem, Belgium). Basophils were gated as IgE-positive cells and analyzed for CD63 expression. The percentage of basophils expressing CD63 and the area under the curve (AUC) are presented. AUC is defined as the integral of the best fit curve: basophil reactivity (% CD63) = F(x) µg/mL venom, where x is the allergen concentration and is calculated for all wasp venom concentrations used in the test.

### 2.4. Treatment

VIT was performed with the use of two types of vaccines with aqueous solutions of wasp venom: Venomenhal (120 µg of wasp venom/via, Hal Allergy, Leiden, The Netherlands) and Diater (Madrid, Spain). The ultra-rush protocol was carried out with a 0.1 μg dose of venom administered subcutaneously, and then 1, 10, 20, 30, and 40 μg at 30-min intervals. After 2 weeks, the patient obtained a maintenance dose of 100 µg (40 µg + 60 µg between 30 min) and 100 µg every 4–6 weeks. This protocol was independent of the type of vaccine used and age group.

### 2.5. Statistical Analysis

The data analysis was based on assessing the differences between groups using the Kruskal–Wallis test for descriptive analysis (Staitstica 8.1, StatPol, Cracow, Poland). The differences in adverse reactions were evaluated using multiple analysis with an ANOVA test. The changes in basophil activation, as indicated by the area under the curve (AUC), and changes in the expression of CD63 were analyzed using the Mann–Whitney U test. A value of *p* < 0.05 was considered as significant.

## 3. Results

### 3.1. Safety

A total of 113 patients with confirmed wasp allergies, including 51 people over 60 years of age (Group A) and 62 young people (18–35 years of age), ultimately qualified for VIT. A total of 111 patients completed a 6-month course of VIT, and during immunotherapy with the ultra-rush protocol, no systemic reactions were observed during the initiation of therapy in any of the studied subgroups (Table 2). At the same time, single large local reactions (>10 cm diameter) were noted in all subgroups, with a slight predominance in the subgroup of young desensitized Diater, but the difference was not statistically significant. During maintenance VIT, two first-degree systemic reactions (according to the Muller scale) were observed in the Diater subgroup of young patients. At the same time, single large local reactions were observed, but they did not dominate any of the analyzed subgroups.

No other complications were observed in the older patients, showing the impact of VIT on the direct cause of decompensation of comorbidities and the need for a significant change in the treatment of cardiovascular diseases and/or diabetes and/or COPD or asthma. In two seniors, the dosage of the initial antihypertensive drug was changed (reduction) during the year of treatment, but this was not directly related to VIT.

None of the patients needed to use epinephrine during the time of observation.

### 3.2. Efficacy

**Figure 2:**
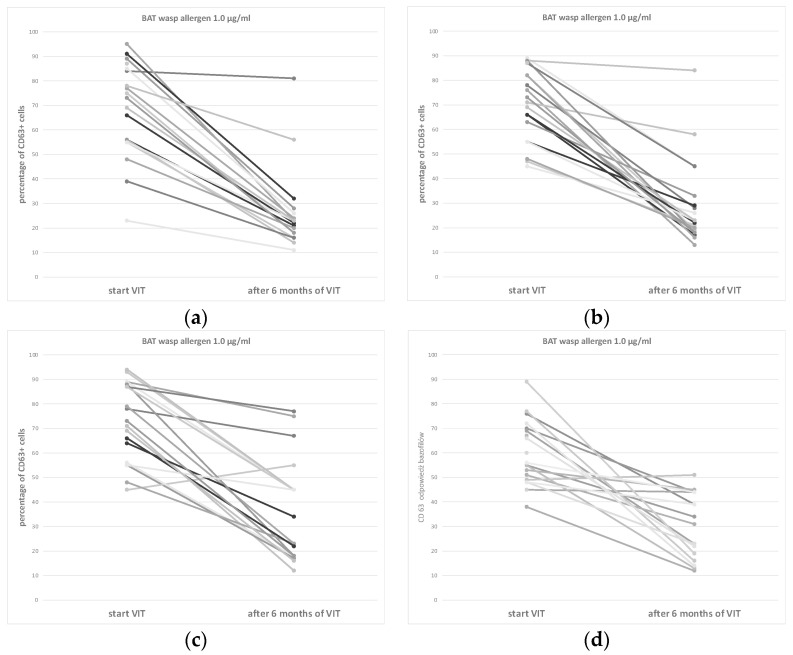
BAT in the subgroups at the start of and after 6 months of VIT: (**a**) older patients on Venomenhal, (**b**) older patients on Diater, (**c**) young patients on Venomenhal, and (**d**) young patients on Diater. VIT: immunotherapy to venom. BAT: Basophil activation test, which was performed with different concentrations of wasp venom extract; however, only data for 1.0 μg/mL were presented for each group, as changes in two points were observed—percentage of basophiles expressing CD63+ and significant depression after 6 months of VIT—in most patients in each group. Different line colours apply to individual patients.

The efficacy of VIT was tested using BAT. There was a statistically significant reduction in CD63 reactivity in 86% or 88% of older people (for Venomenhal and Diater, respectively, *p* > 0.05) and a significant reduction in CD63 reactivity in 84% or 87% of young people (for Venomenhal, Diater, *p* > 0.05). The data are shown in Figure 2a–d. There were no significant differences between age groups or types of vaccines. In the remaining analyzed patients, nonspecific BAT results (in 6–8% of patients) were obtained, or there was no significant improvement compared to the initial examination (3–4% of patients). These results occurred in all subgroups in a similar distribution without favouring subgroups.

**Figure 3:**
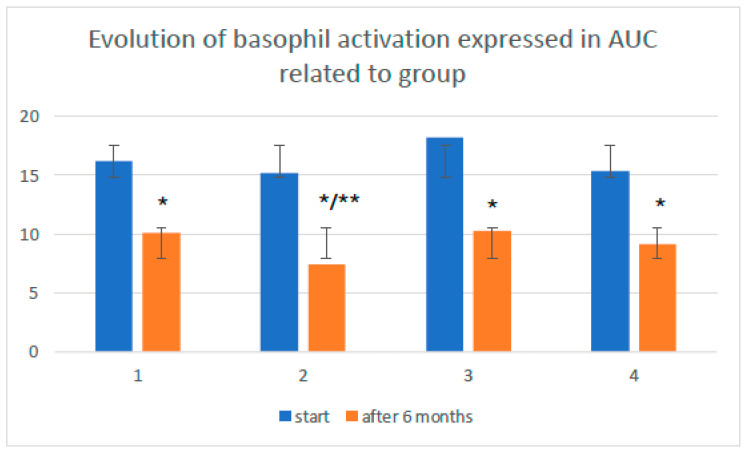
Evolution of basophil activation expressed as the area under the curve (AUC). Legend: Evolution of basophil activation expressed in the AUC related to subgroups: A1—older patients desensitized with the Diater vaccine; A2—older patients desensitized with the Venomenhal vaccine; B1—young patients desensitized with the Diater vaccine; B2—young patients desensitized with the Venomenhal vaccine. *—significant decrease in mean AUC value after 6 months of desensitization; **—significantly greater decrease in mean AUC compared to other subgroups 1, 3, and 4 for *p* < 0.05.

The mean AUC was initially not significantly greater in the subset of older patients desensitized to Venomenhal than that in the remaining patients (*p* = 0.11). After 6 months of treatment, the mean AUC values decreased significantly in all subgroups (*p* < 0.05). The greatest decrease was recorded in the subgroup of older people desensitized with the Diater vaccine, for which the difference was statistically significant (*p* = 0.043). The results are presented in Figure 3.

**Table 2:** Number of adverse reactions, local reactions, and systemic reactions (according to the Mueller scale) during the induction and first 6-month maintenance phases of ultra-rush VIT in different subgroups of study patients.

Type of Adverse Reaction	Older Patients (A)		Young Patients (B)		*p*
	Venomenhal (A1) (*n* = 29)	Diater (A2) (*n* = 28)	Venomenhal (B1) (*n* = 28)	Diater (B2) (*n* = 26)	
Large local reactions *	0.062 ^^^	0.073	0.069	0.075	0.04
Systemic reactions *					0.001
I	0	0	0	0.006 ^^^^	
II	0	0	0	0	
III	0	0	0	0	
IV	0	0	0	0	

VIT—venom immunotherapy. Notes: * Per injection performed in dedicated subgroups. ^^^ Significantly less frequent large local reactions in the group of elderly patients desensitized to Venomenhal than in the other groups (*p* < 0.05). ^^^^ Significantly more frequent first-degree systemic reactions in young patients desensitized to Diater than in the other subgroups (all reactions occurred in the maintenance phase of immunotherapy) (*p* < 0.05).

## 4. Discussion

In general, VIT is safe, but adverse reactions can occur during therapy [14,15]. Based on the obtained results, all of the analyzed ultra-rush protocols are safe. There were only two mild systemic reactions in young patients and no reactions in older patients. This finding is consistent with previous studies [16,17].

To our knowledge, this is the first study assessing the effectiveness of VIT against wasp stings in an age-homogeneous group of older patients. During the induction and maintenance of VIT, no significant differences were observed between the groups of older and younger patients in terms of safety, and the number of large local reactions was consistent with data from the literature. Moreover, the observed single mild systemic reactions in young people are not unexpected and are consistent with the literature [15,17,18]. It is worth emphasizing that these reactions occurred during supportive treatment. It seems that the widely recognized ultra-rush protocol is also safe for older patients, as evidenced by the results presented here. Moreover, a large group of patients over 60 years of age take additional medications, often beta blockers or converting enzyme inhibitors, which in the past were considered risk factors for adverse reactions during VIT [9,10]. Currently, according to research, such drugs do not increase the risk in patients undergoing VIT. However, the greatest benefit from the observation is the confirmation of the effectiveness of VIT within a short period of therapy. It appears that the ageing process of the immune system did not play a significant negative role in the development of tolerance to wasp venom. This is evidenced by the high reactivity of lymphocytes stimulated with wasp venom allergen in the BAT test in the initial tests and a visible reduction in this reactivity after 6 months, which is an indication of the effectiveness of the treatment. BAT is a valuable and proven method for assessing the effectiveness of VIT. In the presented work, it was used as the only indicator for assessing the conducted VIT. The obtained BAT results are comparable to those in young patients and consistent with data in the literature regarding young patients [13,19].

At the same time, IDT results and allergen-specific IgE tests were not significantly different depending on the age group. This may emphasize the value of insect venom diagnostics in groups older than 60 years. The question of the immune system ageing and the efficacy of allergen immunotherapy in older patients is still open. AIT is possible in older patients, and some observations have confirmed it, but not for VIT [20,21]. The aging process of the immune system may significantly influence the ability of the immune system to respond to new allergens and simultaneously increase the accumulation of memory T cells, decrease the number of CD8(+)CD28(+) cells as well as CD4(+), increase the number of CD8(+) CD28(–) apoptosis-resistant cells, and also increase the ratio of CD4/CD8 T cells [22,23]. Additionally, shortening the survival time of immunocompetent cells, reducing their proliferation induced by mitogens, and reducing the number of B lymphocytes is observed in seniors, as well as a reduced proliferative response to mitogenic stimuli, and severely reduced B-cell numbers. These quantitative changes do not significantly impact the induction of allergen tolerance with AIT [22,23].

A significant limitation of these observations is the use of VIT for the analysis of only wasp venom. This is because the group of people allergic to bee stings in our area is much smaller, but a bee sting-allergic group is being collected, and perhaps it will be possible to make similar observations. Another limitation is the relatively small sample size and short observation period. However, the high effectiveness of VIT after 6 months of treatment is visible in the present study, and other authors also present such evidence. The number of BAT marking points during the tests was limited to a minimum for ethical reasons (blood collection).

Finally, it is worth emphasizing that the results of the intradermal tests and the levels of IgE in the blood serum in the presence of wasp venom did not differ significantly between the older and younger groups. This allows us to assume that the reliability of this diagnosis is largely independent of age, i.e., the ageing process of the immune system and skin. The lack of such comparative observations in the field of insect infestation diagnostics does not make it possible to discuss the results. However, in the case of skin tests for inhalant allergens in older people, the reliability of these tests is questionable [24].

## 5. Conclusions

The rapid aqueous vaccine solution protocol for immunotherapy involving wasp venom is an effective and safe treatment method for patients over 60 years of age, as well as for young patients. It seems that the ageing process does not significantly affect the effectiveness of VIT, which requires further research.

## Figures and Tables

**Table 1:** Characteristics of the study patients who completed a 6-month course of immunotherapy (induction and maintenance VIT).

Type of Adverse Reaction	Older Patients (A)		Young Patients (B)		*p*
	Venomenhal (A1) (*n* = 29)	Diater (A2) (*n* = 28)	Venomenhal (B1) (*n* = 28)	Diater (B2) (*n* = 26)	
Median age (range)	68 (61–79)	66 (60–75)	23 (18–33)	25 (18–35)	0.04
Female, *n* (%)	11 (38)	10 (36)	9 (32)	9 (35)	
Number of insect sting systemic reactions acc. Muller (%) prior VIT:					
I	3.2	2.1	4.8 *	2.2	0.02
II	14.5 **	10.9	9.1	11.8	0.01
III	38.9	40.3	35.2	36.7	>0.05
IV	24.8	22.1	21.9	28.9 ^^^	0.03
Average number of injections during observation	14	14.5	13.5	14	NS
% of patients using beta antagonists	25	23	2 ^#^	3 ^#^	0.01
% of patients using inhibitors of ACI	31	29	2 ^#^	6 ^#^	0.01
Mean total IgE serum concentration kU/L ± SD	95.1 ± 20.1	82.1 ± 32.1	88.2 ± 56.2	115 ± 21.5	NS
IDT mean score	4.4	5.1	4.9	4.2	NS
IgE against wasp venom extract kU/L ± SD	16.34 ± 11.2	13.34 ± 8.2	14.9 ± 7.33	15.3 ± 6.6	NS
rVes v1 kU/L ± SD	8.1 ± 2.51	6.01 ± 4.2	2.7 ± 1.4 ^##^	6.45 ± 1.08	0.03
rVes v5 kU/L ± SD	10.58 ± 4.28	8.31 ± 3.02	11.59 ± 3.11	9.11 ± 2.29	NS

Abbreviations: NS: statistically insignificant; SD: standard deviation, and VIT: venom immunotherapy. Notes: * I-degree systemic reactions were significantly more frequent in the group of young patients desensitized by Diater (*p* < 0.05). ** II-degree systemic reactions were significantly more frequent in the group of elderly patients desensitized by Venomenhal (*p* < 0.05). ^ Significantly higher frequency of severe adverse systemic reactions in group B2 (*p* < 0.05)*.^#^* These drugs were used significantly less frequently in the group of young patients than in the group of older patients (*p* < 0.05). ^##^ Significantly lower concentrations of IgE to r Ves v1 were detected in Group B1 (*p* < 005).

## Data Availability

The data presented in this study are available in this article.
